# Examining the physical and chemical contributions to size spectrum evolution during the development of hazes

**DOI:** 10.1038/s41598-020-62296-1

**Published:** 2020-03-24

**Authors:** Liyuan Zhang, Junwei Su, Yu Huang, Qiyuan Wang, Renjian Zhang, Yunfei Wu, Yue Zhang, Yan Cheng, Yuanping He, Shuncheng Lee, Chuck Yu, Zhaolin Gu

**Affiliations:** 1 School of Environmental Science and Engineering, Chang’an University, Xi’an, China; 2 School of Human Settlements and Civil Engineering, Xi’an Jiaotong University, Xi’an, China; 3 Institute of Earth Environment, Chinese Academy of Sciences, Xi’an, China; 4 Institute of Atmospheric Physics, Chinese Academy of Sciences, Beijing, China; 5 School of Faculty of Construction and Environment, Hong Kong Polytechnic University, Hong Kong, China; 6 International Society of the Built Environment (ISBE), Milton Keynes, UK

**Keywords:** Atmospheric chemistry, Pollution remediation

## Abstract

China has experienced severe hazes with high concentrations of particulate matter in recent years. The understanding of the size spectrum evolution of submicron particulate matter is critical to making efficient remediation policies to minimize the regional and global environmental impacts from hazes. During a time period of about one month, we monitored five severe haze episodes in Xi’an and four severe haze episodes in Beijing, which were characterized by two distinct kinds of aerosol mass concentration growth processes: accumulative-rise and abrupt-rise. A new method was developed to quantitatively evaluate the physical and chemical contributions to growth processes by analysing the size spectrum evolution data. The results showed that the accumulative-rise processes are governed by primary emissions and the abrupt-rise processes are governed by secondary chemical reactions. The population balance equations (PBE) were used to describe the variation of size spectrum of fine particulate matter, and the respective contributions of the physical aggregation rate and the chemical growth rate. The PBE model is solved using the adjustable direct quadrature method of moments (ADQMOM) to simulate the abrupt-rise process of haze development and to calibrate the contribution of the physical and chemical effects on the size spectrum of aerosol particles.

## Introduction

Severe haze events have been a major concern for atmospheric pollution in winter all over the world, especially in some developing countries such as China, India, Indonesia, and Mexico. The monitored hour mass concentration of fine particulate matter (PM_2.5_) in Northern China and Northern India in the winter during 2013 to 2018 showed that the PM_2.5_ mass concentration often above 250 μg m^−3^, even might attain a value as high as 1000 μg m^−3^. Under such conditions, people’s exposure to the outside environment can do serious harm to human health^1–3^, causing various diseases of the respiratory system or even death^4,5^. In addition, visibility is reduced to less than 100.0 m, causing problems for air flights and car journeys on major roads. Governments are forced to adopt various emergency and long-term measures in response to such serious pollution problems. In addition to the large amount of primary pollutants, in large quantities, the primary polluting gases can become precursors to secondary pollutants^6^. The deterioration in air quality is also accompanied by physical processes, such as flowing, aggregation, and crushing of large amounts of fine particulate matter, as well as chemical processes, such as new particle formation, various secondary reactions^7^. Moreover, block agglomeration in megacities with intensive buildings and discharge sources can worsen the wind diffusion conditions in the urban canopy^8^, prolonging haze events^9,10^.

Frequently occurring severe haze events have attracted a great deal of scientific interest. The reduction in visibility mainly can be attributed to the scattering and extinction of fine particulate matter, which are determined by the size spectrum, the chemical constituents of particles and the ambient relative humidity^11,12^. During the formation and development of a haze event, the mass concentration of fine particulate matter increased rapidly, accompanied with the rise of the proportion of the secondary matter, illustrating the great contribution of chemical effects^7,13^. In recent five years, Wang, Zheng and other scholars have developed the mechanism of heterogeneous reactions of sulfur oxides and nitrogen oxides on particle surface, and confirmed the significance of atmospheric water vapor and solar radiation on the formation of haze^14–16^. These ambient physical properties not only affect the chemical reaction and PM growth but also the evolution of particle size spectrum and aerosol optical characteristics.

On the other hand, the particle number concentration was stable, or even declined, indicating a more intensive physical aggregation (coagulation and condensation) of aerosol particles^17,18^. Therefore, how to quantitatively distinguish between the physical and chemical effects becomes important for governments to respond and control severe haze pollution

The dispersion of fine particles in the atmosphere are “polydisperse multiphase flows”, which can be simulated by the Population Balance Equation (PBE) combined with the Eulerian equations of air flow^19^. The PBE model is conservation equations for the mean number density function of aerosol particles, depending on the particle properties, the physical space and time^20^. The PBE model was used in this study to determine the contributions to haze formation of the physical effects (particles aggregation, coagulation and condensation), and the chemical effects (chemical growth rate). The revised quadrature method of moments (QMOM), based on the product-difference algorithm^21^, was used to solve the PBE for describing aerosol dynamics under conditions that include new particle formation, evaporation, coagulation, and growth.

This research focused on two questions:

1. How to calculate the contributions of the physical and chemical effects during severe haze formation based on the monitoring data?
2. How can the evolution of the size spectrum state of fine particulate matter be described and simulated with higher accuracy?

The contributions of the physical and chemical effects were quantitatively analysed by examining the variation of the fine particulate matter size spectrum and the concentration of various atmospheric pollutants during haze formation. The highly accurate modified QMOM method^22^ was used to verify the theoretical contribution of the physical and chemical effects. This study provided further insight into the haze development.

### Experiment and methods

Online environmental monitoring was undertaken from December 9, 2015 to January 17, 2016 in Xi’an, and from January 1, 2014 to January 31, 2014 in Beijing.

The Fast-Mobility Particle Sizer Spectrometer (FMPS, TSI Model 3091) was adopted to measure the size distribution of fine particles on the roof of the west 2^nd^ building and the west 4^th^ building in the Qujiang Campus of Xi’an Jiaotong University (108.995E, 34.225 N), at approximately 20 m above ground level. The data of mean diameter and number concentration were corrected by Scanning Mobility Particle Sizers (SMPS). The campus is located at the southeast region of Xi’an, close to the southern three-ring region, without a significant pollution source nearby.

**Figure 1:**
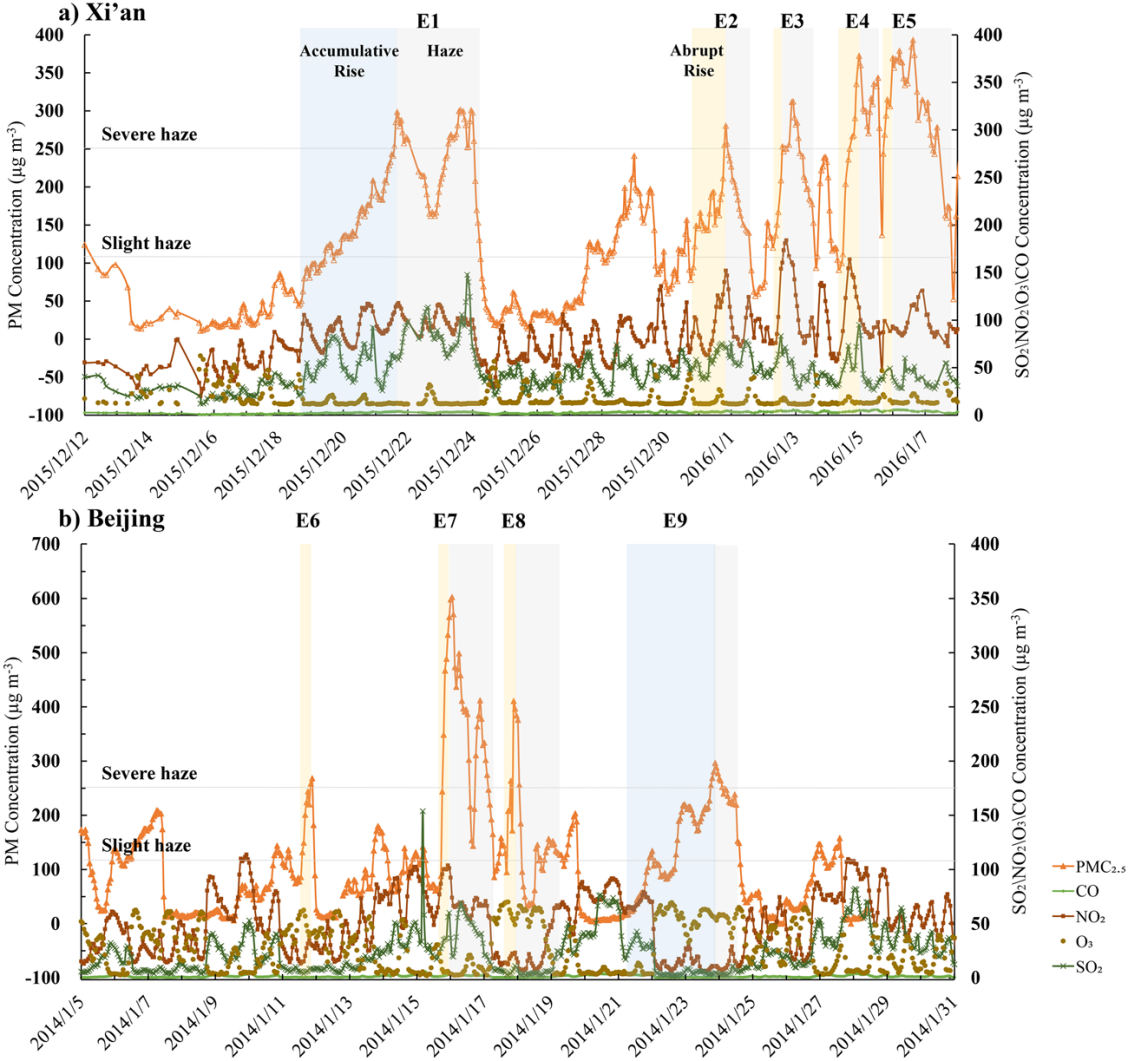
The concentrations of atmospheric pollutants: PM_2.5_, SO_2_, NO_2_ and CO from Dec 12, 2015 to January 7, 2016 in Xi’an (**a**) and January 1, 2014 to January 31, 2014 in Beijing (**b**). All the monitored haze episodes of PM_2.5_ rise processes are depicted in dark grey of which E1 (Xi’an) and E9 (Beijing) illustrate the accumulative-rise processes, and E2-E5 (Xi’an) and E6-E7 (Beijing) illustrate the abrupt-rise processes. The light grey represents the extended period of the haze episode.

The Scanning Mobility Particle Sizers (SMPS) was adopted to measure the size distribution of fine particles on a building roof (approximately 10 m above ground level) at the institute of atmospheric physics, Chinese Academy of Science (116.377E, 39.975 N) in Beijing. The lab is located at the north region of Beijing, close to the northern fourth-ring region, without a significant pollution source nearby. All measurements were made in the standard time period of one hour. The particle distribution monitoring data during the haze episodes in two cities showed that the SMPS and FMPS have similar trends, as shown in Fig. 1.

The Fast-Mobility Particle Sizer Spectrometer (FMPS, TSI Model 3091) can be used to measure the mobility diameter of particles^23^. The instrument consists of two concentric cylinders (classification columns), a corona diffusion charger, and 32 electrometers. The size spectrum data of the fine particulate matter is monitored by the Fast Mobility Diameter in a continuous way, with a detected diameter range of 5.6–560.0 nm, and with a sampling interval of 5 minutes. The FMPS readings were adjusted by factors derived from an intercomparing with a TSI Scanning Mobility Particle Sizer (consisting of a TSI DMA 3081 and a CPC 3772)^24,25^. In this study, the mass concentration of particles with a diameter of 5.6–560.0 nm is denoted as PM_0.6_.

The SMPS is the most commonly used particle sizing instrument, a combination of a Differential Mobility Analyzer (DMA, Model 3081, TSI) and a Condensation Particle Counter (CPC, Model 3772, TSI). In the experiment in Beijing, the detection diameter range is 15.1–661.0 nm, with a sampling interval of 3 minutes. The mass concentration of 15.1–661.0 nm is denoted as PM_0.7_.

Meteorological data, including the ground surface wind speed, precipitation, temperature, air pressure, humidity, and solar radiation were provided by the Qujiang meteorological station at Xi’an and the Haidian meteorological station at Beijing, which belong to the Chinese official meteorological monitoring station system.

The monitored atmospheric pollutant concentrations included PM_2.5_, PM_10_, SO_2_, NO_2_, O_3_, and CO. The effective data values were calculated by averaging the four closest official environmental monitoring stations. The monitored average values for Xi’an were based on Xingqing Community Monitoring Station (34.2629 N, 108.993E), Qujiang Cultural Industry Group Monitoring Station (34.1978N, 108.985E), Xiaozhai Monitoring Station (34.2324 N, 108.94E), and the Textile City Monitoring Station (34.2572 N, 109.06E). The monitored average values for Beijing were based on West Wanshou Nishinomiya Monitoring Station (116.366E, 39.867 N), Wanliu Monitoring station (116.315E,39.993 N), Guanyuan Monitoring Station (116.361E, 39.943 N), and Gucheng Monitoring Station (116.225E,39.928 N). Automated quality control checks on each air pollutant were performed to remove repeated values, overly frequent values, and implausible zeroes. Common signs of missing measurements were replaced with a placeholder. Collectively, these quality control checks excluded 8% of the reported monitored values from the recorded data. The most common exclusion criterion (50% of exclusions) was for pollutants reported to have a continuous non-zero concentration for several hours in a row.

## Results and discussion

### General characteristics of severe haze formation of northern cities of China in winter

During the observation period, the average PM_2.5_ mass concentrations, denoted as PM_2.5_, were 137.2 μg m^−3^ (Xi’an) and 108.1 μg m^−3^ (Beijing), and the highest concentrations were 393.3 μg m^−3^ and 602.7 μg m^−3^, respectively. According to the Air Quality Index (AQI) standard issued by the ministry of environmental protection of China, PM_2.5_ pollutions are classified into four categories: clean (PM_2.5_ < 35.0 μg m^−3^), slightly polluted (35.0 μg m^−3^ < PM_2.5_ < 115.0 μg m^−3^), polluted (115.0 μg m^−3^ < PM_2.5_ < 250.0 μg m^−3^), and heavily polluted (PM_2.5_ > 250.0 μg m^−3^). In the following, we focused on the heavily polluted events, or severe haze episodes.

Figure 1(a) illustrated the five severe haze episodes in Xi’an during the observation period, denoted as E1 from20:00, December 19 to 5:00, December 24, 2015; E2 from 21:00, December 30, 2015 to 11:00,January1, 2016; E3 from 2:00, January 2 to 13:00 January 3, 2016; E4 from 11:00, January 4 to 14:00, January 5, 2016; and E5 from 16:00 to 19:00, January 5, 2016. Figure 1(b) illustrated the three severe haze episodes in Beijing during the observation period, denoted as E6 from 12:00 to 20:00, January 11, 2014; E7 from 17:00 to 23:00, January 15, 2014; E8 from 16:00 to 21:00, January 17, 2014 and E9 from 19:00, January 21 to 21:00, January 23, 2014.

**Figure 2:**
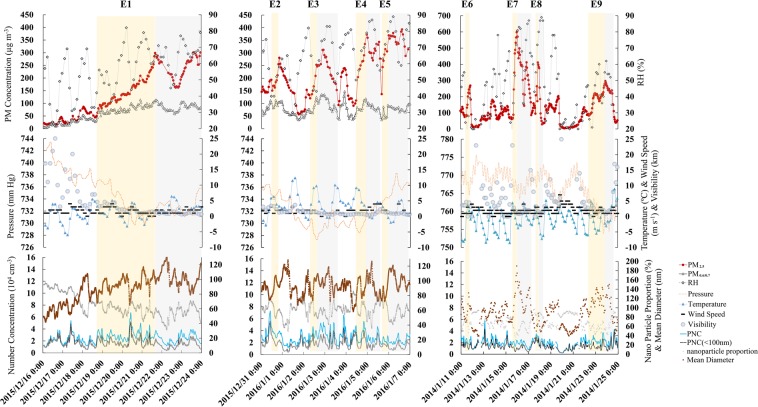
All the atmospheric environmental parameters including ground surface wind speed, precipitation, temperature, air pressure, humidity, visibility, the mass concentration, number concentration, nanoparticle proportion, and MD of aerosol particles for nine haze episodes. All the growth process associated with the monitored haze episodes are depicted in dark grey, and the light grey represents the extended periods of haze. (**a**) shows the accumulative-rise episode E1 in Xi’an; (**b**) shows the four abrupt-rise episodes E2-E5 in Xi’an; (**c**) shows the accumulative-rise episode E9 and the three abrupt-rise episodes E6-E8 in Beijing.

At the early stage of haze episodes, from slight haze to severe haze, for episodes E2-E5 in Xi’an and episodes E6-E8 in Beijing, an abrupt-rise in the PM_2.5_ occurred, with a maximum 5 hours increment of 183.0 μg m^−3^, and an average per-hour increment of 31.0 μg m^−3^. Meanwhile, the concentration of NO_2_ and SO_2_ showed a stable rising trend, with a low ozone and CO concentration in the two cities. Figure 2 shows the meteorological and pollutants parameters of local atmospheric environment during these severe haze episodes. Generally, the low and stable atmospheric boundary layer (less than 300 m) goes against the diffusion and transport of the air pollutants. In addition to the feature of extremely high pollution concentration, most severe haze episodes were formed within a very short period (mostly less than 8 hours).

In particular, the development of haze during E1 in Xi’an and E8 in Beijing were different from that of abrupt-rise haze episodes. The processes were described as an accumulative-rise in the PM_2.5._ During the 62 hours of the episode, the PM_2.5_ gradually increased with an average increment of only 3.8 μg m^−3^ per hour. At the same time, the other atmospheric pollutant concentrations increased slowly.

**Figure 3:**
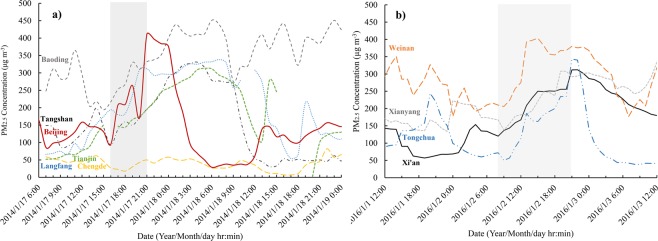
(**a**) The PM_2.5_ mass concentration in Beijing, Baoding, Tianjin, Langfang and Chengde from 6:00, January 17 to 0:00, January 19, 2014. The PM_2.5_ growth process for the haze episode is depicted with grey. b) The PM_2.5_ mass concentration in Xi’an, Xianyang, Tongchuan, and Weinan from 0:00, January 1 to 0:00, January 3, 2016. The PM_2.5_ growth process for the haze episode is depicted with grey.

Based on the monitoring data, all the severe haze episodes E1-E9 were regional pollution events, with a synchronous rise in PM2.5 concentration in cities neighbouring Xi’an and Beijing. Figure 3 illustrate the regional PM2.5 rise in the cities around Beijing and Xi’an.

The growth process of the haze event (E8) in Beijing lasted only 5 hours (16:00–21:00, 17th January). The cities around Beijing, such as Baoding, Tianjin and Langfang showed a synchronous growth trend, with the fastest growth rate and the highest final mass concentration in Beijing. During this period, the dominant wind direction was from the west or northwest, with wind speed of 1.0–2.0 m/s and boundary layer height of 216 m (20:00, 17th January). The diffusion conditions were bad. Within 24 hours before the haze, the dominant wind direction was from the southeast, with wind speed of 2.0–4.0 m/s. Northwest of Beijing is mainly mountainous area, with no obvious pollution sources. The only city, Zhangjiakou (160 km from Beijing), had a PM concentration of 36.0–104.0 μg/m3, which was significantly lower than that of Beijing. Langfang, Tianjin, Tangshan and oceans lie to the southeast of Beijing. Before the occurrence of haze, none of the three cities had experienced high concentrations of PM. Therefore, it was obvious that the main contribution to the formation of this haze event is local source emissions.

The haze (E3) from 2015/1/2 to 2015/1/3 was also a regional pollution. The cities around Xi’an, such as Tongchuan, Xianyang and Baoji showed a synchronous growth trend (9:00–12:00, 2^nd^ January), the wind speed was 0–1.0 m/s, and there was no dominant wind direction (northwest wind, northeast wind and southeast wind all appeared about 3 h). Due to the bad atmospheric diffusion conditions during the formation period of haze, the distance that can affected Xi’an is 40 km, and only one city (20 km to the west of Xi ‘an) is in the area. During PM growth period, the variation characteristics of PM concentration in Xianyang were highly consistent with that in Xi’an. The emission sources of the two cities were similar, mostly located at the junction of the two cities. Therefore, this pollution event in Xi’an mainly came from local sources.

### The evolution and features of diameter spectral distribution during the development of haze

Haze development, even in stable atmospheric conditions, was determined by complicated physical processes and chemical effects, both of which have an influence on the size spectrum of particulate matter. We analysed the evolution of the size spectrum by calculating the total particle number concentration (PNC), particle mass concentration (PM), mean diameter (MD), and nanoparticle proportion (PNC(<100 nm)/PNC(<700 nm)) during the haze event.

**Figure 4:**
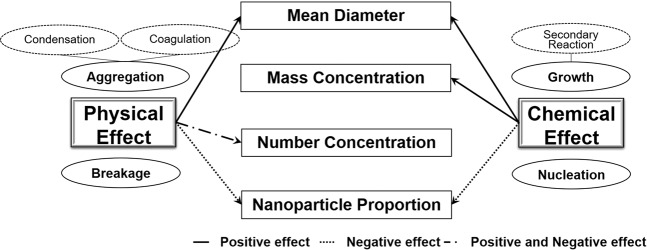
The influence of physical effects and chemical effects on MD, PM, PNC, and nanoparticle proportion during abrupt-rise haze episodes. Both physical and chemical effects contributed to the increase of the average particle size. The PM_2.5_ and PM_0.6_ are determined by chemical effects. The number concentration is mainly determined by physical effects. The nanoparticle proportion is negatively influenced by both physical and chemical effects.

For different types of PM growth processes, abrupt-rise and accumulative-rise, the variations in the PNC, MD, and PM were different, especially the variation in size spectrum. The main reason for different PM growth mechanisms is attributed to the differences in dominance between physical and chemical effects. Figure 4 is a schematic of the physical and chemical effects on aerosol particle growth. The physical effects, including aggregation and breakage of particulate matter, mainly have an influence on the MD and PNC, and the chemical effects, including growth and nucleation of particulate matter, mainly have an influence on the PM and single particle diameter changes^26^. We focused on the abrupt-rise haze episodes to evaluate the contribution of physical effect and chemical effect on the MD, PM, PNC, and nanoparticle proportion by analysing the mass concentration and diameter spectrum data.

In a stable atmospheric environment, the growth of fine particulate matter concentration was caused by primary pollution and secondary chemical reactions. Primary pollution played a leading role in the accumulative-rise haze episodes. According to the observation data and average values reported in the past^27,28^, the contribution from primary pollution to the mass concentration increment was approximately 3.0–5.0 μg m^−3^ per hour in Beijing-Tianjin-Hebei region and Fen-wei region^27,29^. For abrupt-rise haze episodes, there were not only the contribution of primary pollution, but the increase of mass concentration mostly come from secondary chemical reactions, or chemical effects. The variation in mass concentration was almost entirely determined by the chemical growth process. The nucleation was very weak during haze episodes and was ignored in this study. The nanoparticle proportion, the ratio between PNC(100 nm) and PNC(700 nm), can be influenced by both physical effects and chemical effects. The physical effect of aggregation (include condensation and coagulation) resulted in the collision of large amounts of ultrafine particulate matters to produce single particle groups, reducing the number concentration. The reverse occurs, however, during breakage of particle agglomerates. The growth resulting from chemical effects (secondary reaction) enhanced the increase in the diameter of ultrafine particulate matter and reduced the number concentration (<100 nm). According to the monitoring data for E1 and E9, physical effects have a relatively weak influence, and there is a positive correlation between the decline in nanoparticle proportion and the chemical effect strength of MD.

Secondary reactions occurring on the surfaces of particulate matter not only give rise to the mass increase of aerosol particles but also directly lead to the increase of MD of the aerosol particle size spectrum. Therefore, we can estimate the contribution of chemical and physical effect on MD as follows. First, the chemical effect on the increment of MD is defined as:

1 $${\rm{Chemical}}\,{\rm{Contribution}}\,({\rm{MD}})=\frac{{\Delta {\rm{MD}}}_{C}}{{\rm{MD}}({T}_{0})}$$

Where ΔMD_C_ is the increase of MD caused by chemical effects; MD(T_0_) is the MD at T_0_, the start of haze episodes.

Second, the physical effect on the increment of MD is defined as

2 $${\rm{Physical}}\,{\rm{Contribution}}=\frac{{\Delta {\rm{MD}}}_{{\rm{P}}}}{{\rm{MD}}({{\rm{T}}}_{0})}$$

where ΔMD_P_ is the increase of MD caused by physical effects.

Therefore, the sum of the physical and chemical effects on the increment of MD is calculated as

3 $${\Delta {\rm{MD}}}_{{\rm{C}}}+{\Delta {\rm{MD}}}_{{\rm{P}}}={{\rm{MD}}({\rm{T}}}_{1})-{\rm{MD}}({{\rm{T}}}_{0})$$

where MD(T_1_) is the MD at the moment of T_1_, the end of the PM increase periods.

As mentioned above, assuming that the mass concentration increment from the contribution of primary pollution for accumulative-rise processes is *ε* μg m^−3^ per hour, we can obtain

4 $${{\rm{PM}}}_{2.5}({{\rm{T}}}_{1})={{\rm{PM}}}_{2.5}({{\rm{T}}}_{0})+{\Delta {\rm{PM}}}_{{\rm{C}}}+\varepsilon \Delta {\rm{t}}\,$$

where PM_2.5_(T_0_) is the mass concentration of PM_2.5_ at the start moment T_0_; PM_2.5_(T_1_) is the mass concentration of PM_2.5_ at the end moment of T_1_; ΔPM_C_ is the increment of PM_2.5_ caused by chemical effects during the period from T_0_ to T_1_; *ε* is the growth rate of PM_2.5_ determined by physical effects; and Δt is the time duration from T_0_ to T_1_.

The MD is usually calculated using the value of PM_2.5_ at any moment as follows.

5 $${{\rm{PM}}}_{2.5}=\frac{1}{6}\cdot {{\rm{\pi }}{\rm{MD}}}^{3}{\rm{\rho }}$$

where ρ = 1.2 g m^−3^ is the density of fine particles.

Combining Eqs. (4) and (5), the chemical and physical effects on the increment of MD are calculated by

6 $${\rm{Chemical}}\,{\rm{Contribution}}\,({\rm{MD}})=\frac{{\Delta {\rm{MD}}}_{{\rm{C}}}}{{\rm{MD}}({{\rm{T}}}_{0})}={\left(\frac{{{\rm{PM}}}_{2.5}({{\rm{T}}}_{1})-\varepsilon \Delta {\rm{t}}}{{{\rm{PM}}}_{2.5}({{\rm{T}}}_{0})}\right)}^{\frac{1}{3}}-1$$

7 $${\rm{Physical}}\,{\rm{Contribution}}=\frac{{\Delta {\rm{MD}}}_{{\rm{P}}}}{{{\rm{MD}}({\rm{T}}}_{0})}=1-\frac{{\Delta {\rm{MD}}}_{{\rm{C}}}}{{\rm{MD}}({{\rm{T}}}_{0})}$$

According to Eq. (6) and Eq. (7), for the six abrupt-rise haze episodes in Xi’an and Beijing, the contributions of chemical effects on the increase in MD were 17.4%, 26.0%, 48.8%, 37.0%, 56.1%, and 16.3%, with an average contribution of 33.6%, giving an average contribution of physical effects of 66.4%. For the accumulative-rise haze episodes E1 and E8, the physical effects on the increase of MD was calculated to be 90.9% and 80.4%, respectively, and the chemical effects on the MD increase was only 9.1% and 19.6%.

A linear regression on the chemical contribution to MD was conducted to determine the regression coefficient and the average diameter increase rate. The following equations were derived:

8 $${\rm{MD}}\,{\rm{Increase}}\,{\rm{Rate}}=1.76\cdot {\rm{Chemical}}\,{\rm{contribution}}\,({\rm{MD}})+0.04$$

Substituting Eq. (6) into Eq. (8),

9 $${\rm{MD}}\,{\rm{Increase}}\,{\rm{Rate}}=1.76\cdot \left[{\left(\frac{{{\rm{PM}}}_{2.5}({{\rm{T}}}_{1})-\varepsilon \Delta {\rm{t}}}{{{\rm{PM}}}_{2.5}({{\rm{T}}}_{0})}\right)}^{\frac{1}{3}}-1\right]+0.04$$

**Figure 5:**
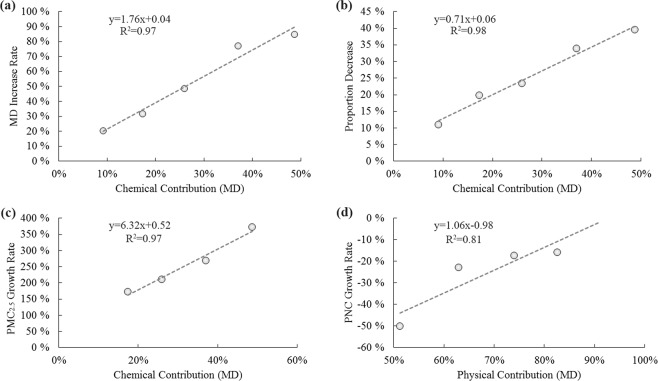
Linear regression analyses of the chemical contribution of MD on the nanoparticle proportion decrease, MD increase rate and PNC growth rate, and physical contribution on the PM_2.5_ growth rate. (**a**) illustrates the correlation between the chemical contribution of MD and the increase rate of the mean diameter. (**b**) illustrates the correlation between the chemical contribution of MD and the decline in nanoparticle proportion. (**c**) illustrates the correlation between the chemical contribution of MD and the PM_2.5_ growth rate. (**d**) illustrates the correlation between the physical contribution of MD and the PNC growth rate.

The correlation between the chemical contribution of MD and the MD increase rate was R^2^ = 0.96, as shown in Fig. 5(a).

The equation of linear regression between the decrease in nanoparticle proportion and the chemical contribution of MD was shown in Fig. 5(b):

10 $${\rm{Proportion}}\,{\rm{Decrease}}=0.71\cdot {\rm{Chemical}}\,{\rm{contribution}}({\rm{MD}})+0.06$$

Substituting Eq. (6) into Eq. (10),

11 $${\rm{Proportion}}\,{\rm{Decrease}}=0.71\cdot \left[{\left(\frac{{{\rm{PM}}}_{2.5}({{\rm{T}}}_{1})-\varepsilon \Delta {\rm{t}})}{{{\rm{PM}}}_{2.5}({{\rm{T}}}_{0})}\right)}^{\frac{1}{3}}-1\right]+0.06$$

The correlation R^2^ = 0.98, was shown in Fig. 5(b).

The equation of the linear regression of the total PM_2.5_ increase rate and the chemical contribution of MD were shown in Fig. 5(c):

12 $${{\rm{PM}}}_{2.5}\,{\rm{Growth}}\,{\rm{Rate}}=6.32\cdot {\rm{Chemical}}\,{\rm{contribution}}\,({\rm{MD}})+0.52$$

The correlation between the chemical contribution of MD and the total PM_2.5_ increase rate was R^2^ = 0.97.

The correlation between the physical contribution of MD and the PNC growth rate was R^2^ = 0.81, as shown in Fig. 5(d), which was calculated by Eq. (7) and Eq. (12).

The four severe haze episodes (E2-E5) in Xi’an, shown in Table 1, demonstrate that the mean diameter of all the size distributions with diameter less than 700 nm increased by an average of 60.6%, but their number concentrations were stable, and even exhibited a slightly declining trend. The growth rate of PM_0.6_ was 73.1% lower than that of PM_2.5_, resulting in a nanoparticle proportion that was reduced by an average 29.2% for these episodes.

**Table 1:** The data of abrupt-rise and accumulative-rise at the beginning (T0) and the end (T1).

City	Abrupt Rise		Accumulative Rise	
	Xi’an	Beijing	Xi’an	Beijing
Duration (hrs)	6.8	7.0	73.0	51.0
PM_2.5_ (T_0_) (μg m^−3^)	130.0	83.0	44.5	75.0
PM_2.5_ (T_1_) (μg m^−3^)	318.5	412.2	298.8	296.7
PM_2.5_ increase per hour (μg m^−3^ h^−1^)	31.0	51.1	3.5	4.4
Particle Number Concentration (T_0_) (10^4^ cm^−3^)	3.8	2.4	1.2	2.4
Particle Number Concentration (T_1_) (10^4^ cm^−3^)	2.8	1.9	3.0	3.6
PNC_0.1_ (Diameter <100 nm) (T_0_) (10^4^ cm^−3^)	3.0	1.7	0.8	1.8
PNC_0.1_ (Diameter <100 nm) (T_1_) (10^4^ cm^−3^)	1.4	1.0	1.6	2.5
Nanoparticle Proportion (T_0_)	78.3%	67.3%	64.0%	73.4%
Nanoparticle Proportion (T_1_)	49.2%	48.7%	53.0%	69.5%
MD Increase Rate	60.6%	61.1%	20.2%	12.2%
Chemical contribution (MD)	32.3%	36.2%	9.1%	19.6%
Physical contribution (MD)	67.7%	63.8%	90.9%	80.4%

For severe haze episodes E6 and E7 in Beijing, shown in Table 1, the mean diameter of all the size distributions with diameter less than 661 nm increased by an average of 61.1%, but, again, the number concentration was stable, even exhibiting a slightly declining trend. The growth rate of PM_0.7_ was 416.1% lower than that of PM_2.5_, resulting in a nanoparticle proportion that was reduced by an average of 39.5% for the 2 episodes.

By comparison, for accumulative-rise haze episodes E1 in Xi’an and E8 in Beijing, the mean diameter only increased by an average of 20.2% (E1) and 12.2% (E8), respectively, but the number concentration was relatively stable. The increases in PM_0.6_ (E1) and PM_0.7_ (E8) were 243.6% and 247.2%, respectively, which are less than the increase in PM_2.5_, resulting in a nanoparticle proportion that was reduced by 11.0% (E1) and 3.9% (E8).

The long term accumulative-rise process was mainly the accumulation of relatively large particulate matter from primary pollution sources. The PM growth in the increment of PM_0.6_ in Xi’an or PM_0.7_ in Beijing was much slower than PM_2.5_. For the abrupt-rise process, with a rapidly growing PM_2.5_, the ultrafine particles showed a significantly accelerated growth with aggregation features, which form relatively large particles rapidly. The proportion of the number concentration of nanoparticulate matter reduced, and the mean diameter also reduced rapidly.

### PBE simulation of the distribution of fine particles during the abrupt-rise period

According to the results of the above analysis, the relative strength of the physical and chemical effects can have a significant influence on size spectrum evolution during the abrupt-rise period. Both the rate of increase in MD and the rate of decline in the nanoparticle proportion have an approximately linear relation with the chemical effect contribution. PM is totally determined by chemical effects, and PNC is mainly influenced by physical effects. Such a result can be further verified by the variation in particle size spectrum described by the PBE.

To consider micro-interactions and the size spectrum of particles in computational simulations with multiphase flows, the PBE was used as a good method to describe the dispersal phase.

The PBE for systems with continuous operations are formulated as^22^:

13 $$\frac{\partial {\rm{f}}({\rm{V}};{\rm{x}},{\rm{t}})}{\partial {\rm{t}}}+\frac{\partial }{\partial {{\rm{x}}}_{{\rm{i}}}}( < {{\rm{u}}}_{{\rm{i}}}\,{ > }_{{\rm{V}}}{\rm{f}}({\rm{V}};{\rm{x}},{\rm{t}}))={\rm{S}}({\rm{V}})$$

14 $$\frac{\partial {\rm{f}}({\rm{V}};{\rm{t}})}{\partial {\rm{t}}}+\frac{{\rm{f}}({\rm{V}};{\rm{t}})-{\rm{f}}{({\rm{V}})}_{{\rm{in}}}}{{\rm{\tau }}}={\rm{S}}({\rm{V}})$$

where V represents the volume of particle referred to as the internal coordinate; t represents time; f(V; t) represents the number density function, representing the particle size spectrum at the outlet; f(V)_in_ represents the number density function at the inlet; τ represents the relaxation time; and S(V) represents the source term that was used to describe the micro-behaviour.

The aggregation (condensation and coagulation) of particles is formulated as^22^:

15 $${\rm{S}}({\rm{V}})=\frac{1}{2}{\int }_{0}^{{\rm{V}}}{\rm{\beta }}({\rm{V}}-{\rm{v}},{\rm{v}}){\rm{f}}({\rm{V}}-{\rm{v}};{\rm{t}}){\rm{f}}({\rm{v}};{\rm{t}}){\rm{dv}}-{\rm{f}}({\rm{V}};{\rm{t}})\,{\int }_{0}^{\infty }{\rm{\beta }}({\rm{V}},{\rm{v}}){\rm{f}}({\rm{v}};{\rm{t}}){\rm{dv}}$$

where β(V, v) represents the aggregation kernel between one particle (volume is V) and another particle (volume = v); $\frac{1}{2}{\int }_{0}^{{\rm{V}}}{\rm{\beta }}({\rm{V}}-{\rm{v}},{\rm{v}}){\rm{f}}({\rm{V}}-{\rm{v}};{\rm{t}}){\rm{f}}({\rm{v}};{\rm{t}}){\rm{dv}}$ represents the addition of particles (volume = V) due to the aggregation of smaller particles; and $-{\rm{f}}({\rm{V}};{\rm{t}}){\int }_{0}^{\infty }{\rm{\beta }}({\rm{V}},{\rm{v}}){\rm{f}}({\rm{v}};{\rm{t}}){\rm{dv}}$ represents the loss of particles (volume = V) due to the aggregation with other particles.

The breakage of particles is formulated as^22^:

16 $${\rm{S}}({\rm{V}})={\int }_{{\rm{V}}}^{\infty }{\rm{a}}({\rm{v}}){\rm{b}}({\rm{V}}|{\rm{v}}){\rm{f}}({\rm{v}};{\rm{t}}){\rm{dv}}-{\rm{a}}({\rm{V}}){\rm{f}}({\rm{v}};{\rm{t}})$$

where a(V)representsthe particle breakage kernel conditioned on volume V; b(V | v) represents the daughter distribution; ${\int }_{{\rm{V}}}^{\infty }{\rm{a}}({\rm{v}}){\rm{b}}({\rm{V}}|{\rm{v}}){\rm{f}}({\rm{v}};{\rm{t}}){\rm{dv}}$from Eq. (14) represents the addition of particles (volume = V)due to the breakage of larger particles; and ${\int }_{{\rm{V}}}^{\infty }{\rm{a}}({\rm{V}}){\rm{f}}({\rm{v}};{\rm{t}})\,$is the loss of particles (volume = V) due to the breakage.

The growth process is formulated as^22^:

17 $${\rm{S}}({\rm{V}})=-\frac{\partial }{\partial {\rm{V}}}[{\rm{G}}({\rm{V}}){\rm{f}}({\rm{V}};{\rm{t}})]$$

where G(V) represents the growth rate of particles with volume V. This is determined by a specific physical or chemical process, as well as features of the environmental fluid and the dispersed phase entity, which needs to be obtained from the fluid equation and the reaction equation.

Moment methods were the first numerical method to address the PBE^30^. The quadrature method of moments (QMOM) is used to simplify the PBE theory with volume as the internal coordinate:

18 $${{\rm{m}}}_{{\rm{k}}}({\rm{t}})={\int }_{0}^{\infty }f({\rm{\xi }};{\rm{t}}){\xi }^{k}{\rm{d}}{\rm{\xi }}$$

Moments with different values of k represent different physical meanings. If *ξ* represents the characteristic length, then m_0_represents the number of particles and m_1_represents the total length of particles. k_s_m_2_ represents the total area of particles and k_v_m_3_represents the total volume of particles. K_v_ and k_s_ represent the volume and shape factors for the area.

Marchision and Fox detected two problems with the QMOM when implemented using a CFD code^31^: (1) when there is a strong dependency between the dispersed-phase velocity and the internal coordinates (e.g., fluidized bed and bubble column modelling), the solution of the transport equations is difficult to treat as a system; and (2) in the case of multivariable conditions of PBEs^32^, use of the QMOM becomes difficult because of the high computing demands, especially for CFD applications. Therefore, the direct quadrature method of moments (DQMOM) was proposed to reduce the demand on computational resources and to extend it to multivariable cases^33^.

Choosing the best adjustable factor based on the true distribution is very important but difficult. The adjustable direct quadrature method of moments (ADQMOM) was proposed based on the DQMOM to solve the problem of choosing the best adjustable factor^34^. A novel procedure for selection of the optimal adjustable factor is also included in the ADQMOM.

We adopt the ADQMOM to simulate the variation in fine particle distribution during the period of haze formation to verify the influence of physical and chemical effects.

The result of the calculation can be influenced by multiple factors:

1. The aggregation rate and breakage rate are influenced by physical effects, and the growth rate in the PBE equation is influenced by chemical effects.
2. The initial size spectrum could have a significant influence on the result.

**Figure 6:**
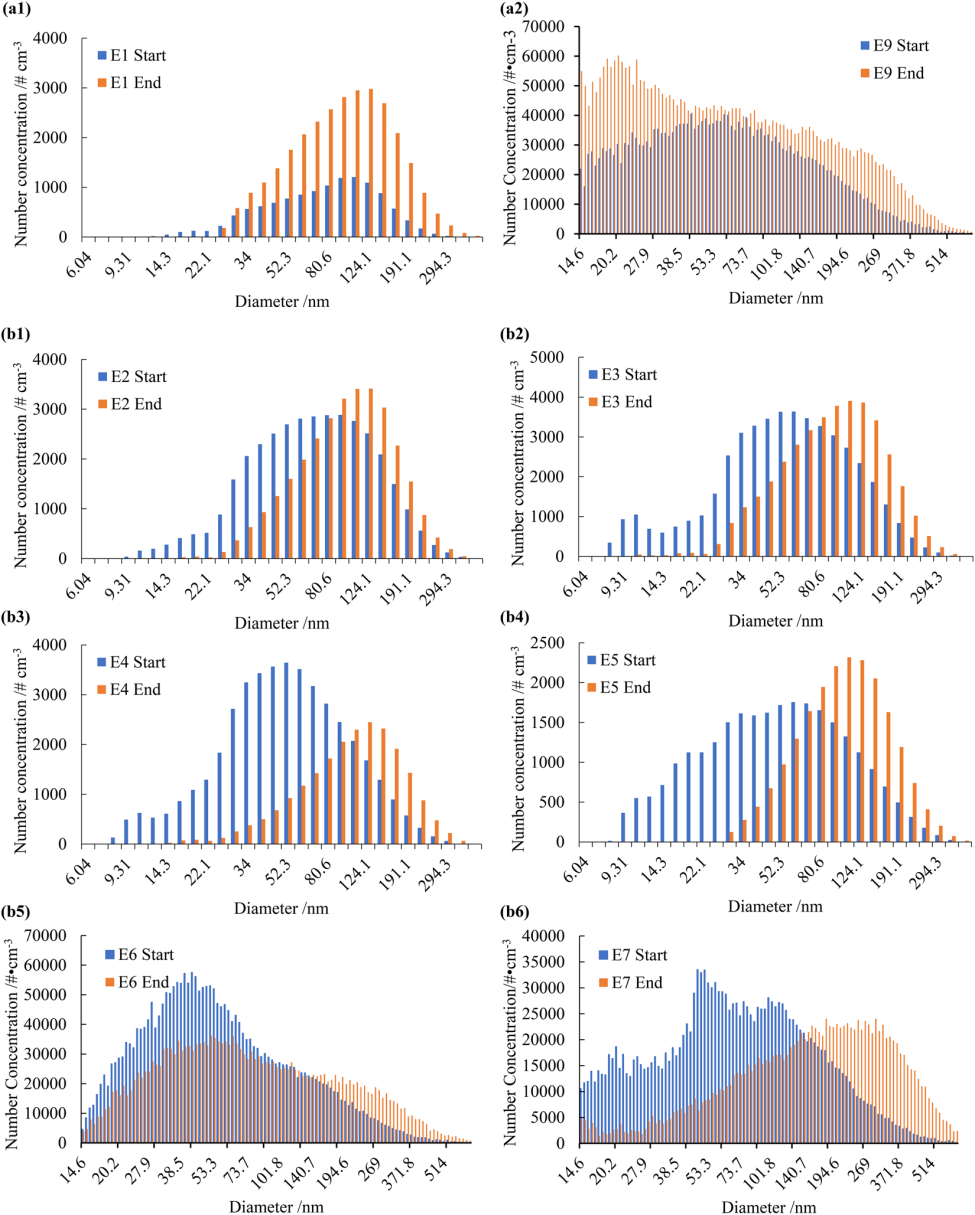
Particle distribution at the starting and the ending moments in two accumulative-rise episodes (**a1**–**a2**) and six abrupt-rise episodes (**b1**–**b6**) in Xi’an and Beijing.

The distribution state during the accumulative-rise process differed greatly from the abrupt-rise, as shown in Fig. 6(a1,a2). During the six abrupt-rise processes, there were large differences between the initial distribution states (R^2^ = 0.9096), but similar final distribution states (R^2^ = 0.9798), as shown in Fig. 6(b1–b6). During the abrupt-rise process, there was not a large variation in the number concentration, and the strong chemical effect produced a significant variation in mass concentration. During the accumulative-rise process, however, there was significant growth in the number concentration (145%) for all particle spectrums, indicating that the increased number of particles neither comes from nucleation nor the aggregation of nanoparticles. Instead, the increase in the PNC and PM was mainly due to the accumulation of primary pollution. The PBE equation would not be applicable to the accumulative growth process because it is difficult to evaluate and introduce new particulate matter in the calculation.

The paragraphs above analyse the strength of physical and chemical effects in different increase events. The growth rate in the PBE equation was adjusted because of the different chemical contributions (MD) in various growth processes. The aggregation and breakage rates in the PBE equation were adjusted according to the various physical contributions (MD) for the various growth processes.

The relationship between the chemical growth rate (i) and mass concentration growth rate is determined by the following equations.

19 $${{\rm{PM}}}_{2.5}\,{\rm{Growth}}\,{\rm{Rate}}={{\rm{e}}}^{{{\rm{in}}}_{0}{\rm{t}}}-1\,{({\rm{n}}}_{0}=0.033)$$

Substituting this into Eq. (8), to obtain

20 $${\rm{i}}={(1/{\rm{n}}}_{0})\mathrm{ln}(6.32\cdot {\rm{Chemical}}\,{\rm{Contribution}}\,({\rm{MD}})+0.52)$$

The relationship between physical aggregation on rate (k) and PNC growth is

21 $${\rm{PNC}}\,{\rm{Growth}}\,{\rm{Rate}}={{\rm{e}}}^{-(0{\rm{.091k}}+0.0024){\rm{t}}}-1$$

**Figure 7:**
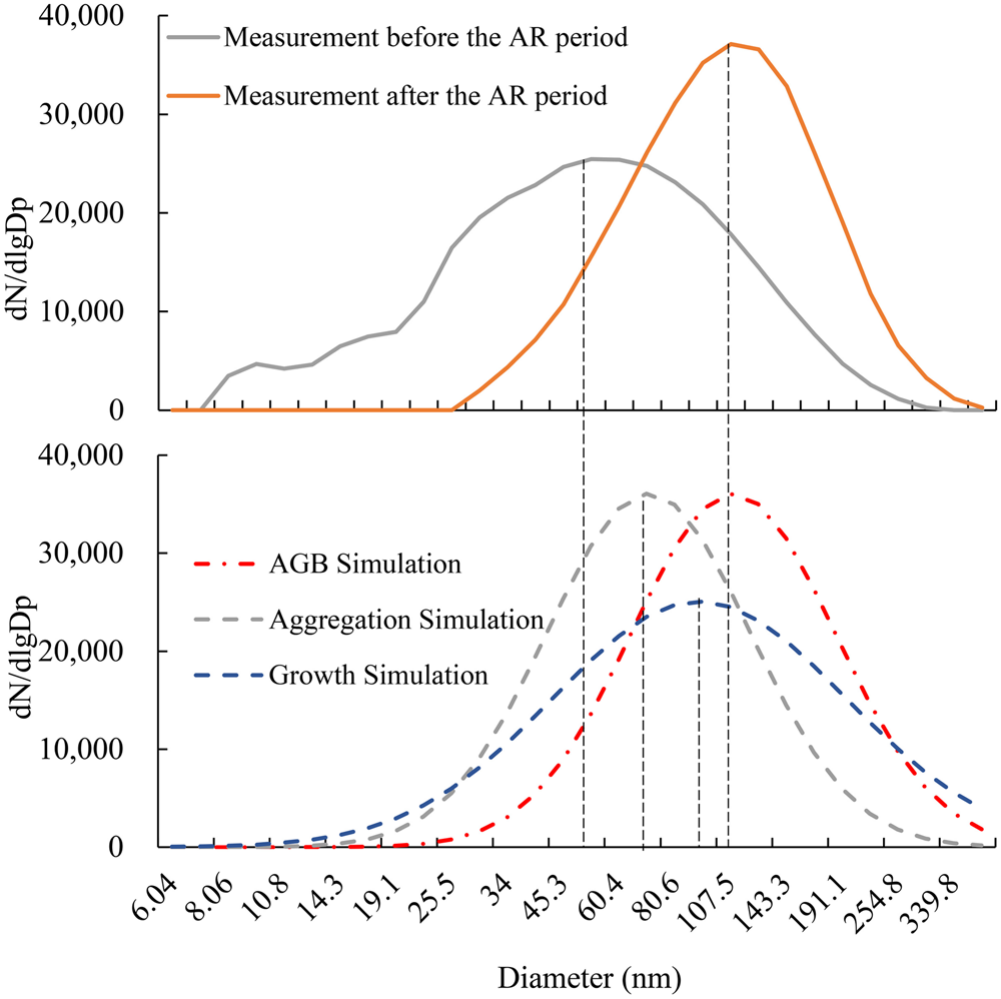
Measurement (**a**) and simulation (**b**) of the change in the particle size distribution during abrupt-rise episode (E3), considering the physical (aggregation simulation model) and chemical conditions (growth simulation model), and the combination of both (AGB simulation model).

Figure 7(a) illustrates the average distribution before and after the abrupt-rise period. During the abrupt-rise process, the average growth rate in mass concentration was 24.2%, which was calculated as i = 6.45. The average growth rate of the number concentration was −4.0%, which was calculated as k = 0.42.

Substitute the above coefficients into the PBE equation set, which distinguishes chemical from physical effects, as shown in Fig. 7(b), to obtain the result. We simulate the Episode 3 case (abrupt rise) from 2:00, January 2 to 13:00 January 3, 2016 in Xi’an. During the 11-hours period, PM_2.5_ increased from 76.0 μg m^−3^ to 253.2 μg m^−3^, mean diameter (<560 nm) increased from 63.8 nm to 114.6 nm. Three models were adopted to simulate the variation of size spectrum during this abrupt rise episode.

According to the simulated result to the diameter spectrum, the chemical contribution mainly influences the growth of the MD, and physical contribution mainly influences the variation in PNC, which is in consistent with the theoretical analysis results. When only physical effects were considered, the simulation results of aggregation and breakage model showed that the average diameter increased by 26.8%. Meanwhile, the number of particles with smaller size decreased rapidly due to aggregation, and the particle size distribution tended to be concentrated. When only chemical effects were considered, the simulation results of growth model showed that the average diameter increased by 75.3% with the similar standard deviations of original size spectrum. When both of physical and chemical effects were considered, the simulation results of aggregation-growth-breakage (AGB) model showed that the average diameter increased by 93.4% with a more concentrated size distribution.

## Conclusions

The severe haze episodes that occurred in Xi’an from December 2015 to January 2016, and in Beijing from January 1, 2014 to January 31, 2014 were classified as two accumulative-rise haze episodes and six abrupt-rise haze episodes. The average mass concentration increment for accumulative-rise haze episodes was 3.8 μg m^−3^ h^−1^, with a duration of 51 to 73 hours. The average mass concentration increment for abrupt-rise haze episodes was more than 20.0 μg m^−3^ h^−1^, with a duration of 4 to12 hours.

The difference between accumulative-rise and abrupt-rise haze episodes can be attributed to the changing features of the particle size spectrum during haze events, illustrating the difference between the physical and chemical effects on the particle size spectrum during the haze development process.

Based on the monitoring data, the chemical effects on the MD, PM, and nanoparticle proportion was evaluated. For six abrupt-rise haze episodes, the average contribution values of chemical and physical effects on the MD were 32.3% and 67.7%, respectively. For two accumulative-rise haze episodes, the average contribution values of chemical and physical effects on the MD were  14.4% and 85.6%, respectively. It was obvious that in Xi’an and Beijing the contribution of the chemical effect during the abrupt-rise episodes is much more than during accumulative-rise episodes.

The PBE was adopted in this study to describe the size spectrum of fine particulate matter. Based on the experimental data for both Xi’an and Beijing, the PBE is proved to be an appropriate method for calibrating the contribution of the physical and chemical effects, thus improving the simulation accuracy of the abrupt-rise process at the early stage of haze development.

## Data Availability

All data generated or analysed during this study are included in this published article.
